# Factor VII deficiency: a rare genetic bleeding disorder in a 7-year-old child: a case report

**DOI:** 10.1186/s13256-023-03884-3

**Published:** 2023-04-14

**Authors:** Hajaj Mohamed Salum, Joyce Lukumay, Kandi Muze, Peter Swai, Christina Kindole, Honesta Kipasika, Monica Apollo, Lulu Chirande, Francis Furia

**Affiliations:** 1 Department of Paediatrics and Child Health, Muhimbili University of Health and Allied Sciences, Dar es Salaam, Tanzania; 2 Department of Paediatrics, Muhimbili National Hospital, Dar es Salaam, Tanzania

**Keywords:** Factor VII (FVII) deficiency, Bleeding, Rare, Case report

## Abstract

**Background:**

Factor VII deficiency is a rare inherited bleeding disorder that has similar clinical presentation to hemophilia.

**Case report:**

A 7-year-old male child of African origin experienced recurrent nasal bleeding since 3 years of age and recurrent swelling of the joints that was remarkable at the age of 5–6 years. He received multiple blood transfusions and has been managed as a patient with hemophilia until he presented to our facility. Reviewed evaluation of the patient revealed abnormal prothrombin and normal activated partial thromboplastin time, FVII analysis showed activity level of less than 1%, and the diagnosis of FVII deficiency was made. The patient was treated with fresh frozen plasma, vitamin K injection, and tranexamic tablets.

**Conclusion:**

Even though factor VII deficiency is an extremely rare bleeding disorder, it does occur in our setting. This case highlights the need for clinicians to consider this condition when faced with challenging patients presenting with bleeding disorders.

## Background

Factor VII (FVII) deficiency is an extremely rare bleeding disorder with prevalence of 1:500,000 worldwide [1, 2]. FVII deficiency may be inherited as an autosomal recessive disorder or may be acquired as a complication of several conditions including sepsis and malignancies [3, 4]. The autosomal recessive disorder is a result of over 100 mutations, mostly missense mutations in the FVII gene located on chromosome 13 [5]. Two types of FVII deficiency have been described; type 1 deficiencies resulting from decreased biosynthesis or accelerated clearance, and type 2 abnormalities representing a dysfunctional molecule [5]. FVII is one of the vitamin K-dependent factors; therefore, its deficiency may be accompanied by reductions in other vitamin K-dependent coagulation factors including factors IX, X, and prothrombin [6]. Acquired deficiency of FVII that is independent of vitamin K deficiency has been reported [7]. The prevalence of FVII deficiency may be underestimated because of patients who are asymptomatic. The median age for discovery of inherited defect is 8 years, with most patients presenting with epistaxis (60%), gum bleeding (34%), easy bruising (36%), and menorrhagia. Among these, only 10–15% exhibit potentially life-threatening or limb-threatening hemorrhages [1, 8].

In this case report we describe a 7-year-old boy with FVII deficiency, who has family history of bleeding disorder and has lost two siblings due to severe bleeding.

## Case history

A 7-year-old African boy from non-consanguineous parents presented to us with a history of recurrent nose bleeding for 4 years, which was first noted at 3 years of age. The time between bleeding episodes was initially about 6 months and duration of bleeding was 2–5 minutes; the time between episodes was getting shorter and duration of bleeding increased subsequently lasting for more than 30 minutes at the time of presentation to our facility. For the past year, he bled at least once a month and required multiple transfusions; bleeding was confined to the nose only. He was attending his hometown regional hospital where initial investigations showed persistent low hemoglobin, abnormal prothrombin, and normal activated partial thromboplastin time. At the time of presentation to our facility, the patient had recurrent swelling of right elbow, left wrist, and right knee for 1 year, and there was deformity of the right knee, impairing walking.

Patient received all vaccinations according to the Tanzania Expanded Program of Immunization (EPI) without any complications, and had no history of jaundice, other chronic illness, or use of any hepatotoxic drugs. He is the fifth born of five children in the family. The first born, a male, apparently normal, died at the age of 3 weeks following excessive bleeding after removal of natal teeth by traditional healers. The second born, a female, was apparently well until 3 years of age when she developed recurrent bleeding and joint swelling as the index, necessitating multiple blood transfusions. She eventually died at the age of 7 years due to excessive bleeding. The third and fourth born are doing well. The paternal grandfather, who died at the age of 80 years, was reported to have had similar complaints of recurrent bleeding.

**Fig. 1:**
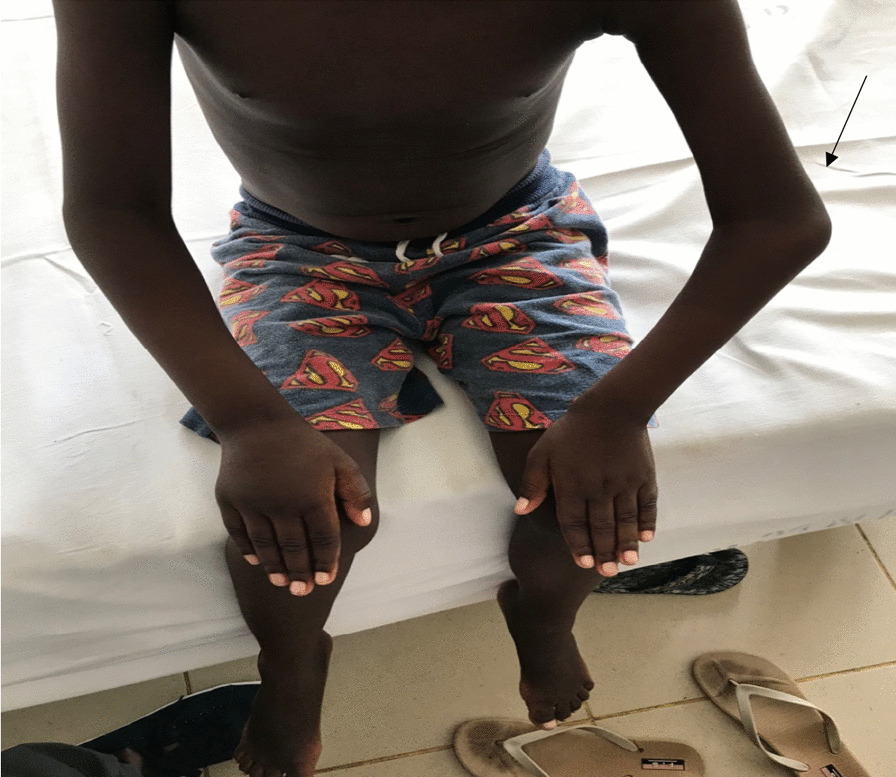
Arrow showing a swollen left elbow

**Fig. 2:**
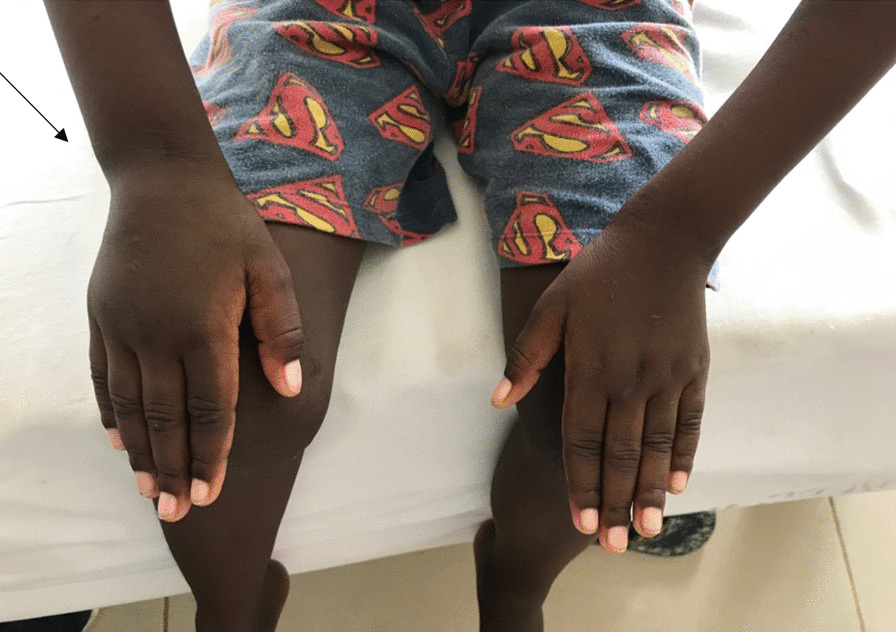
Arrow showing a swollen right wrist

**Fig. 3:**
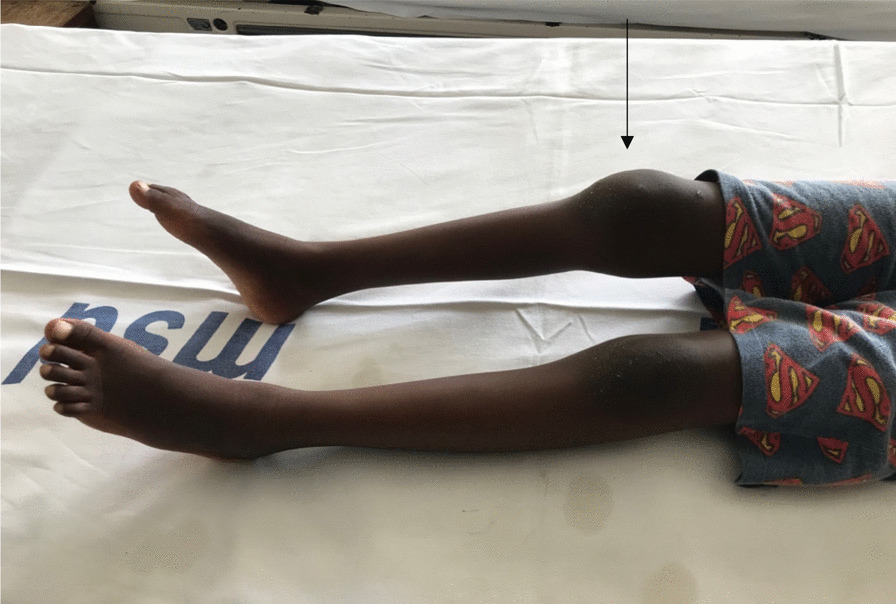
Arrow showing a swollen right knee

On physical examination, the patient was alert, afebrile, with some palmar pallor, however, he had neither jaundice nor dysmorphic features or petechiae. Musculoskeletal examination revealed limping gait with avoidance of weight bearing on the right leg on walking. He had normal abduction of both arms to 180° without wincing, and could touch above T10 with both hands. He could neither extend the elbows to 180°, nor could he flex the wrists to 90°. The left elbow and right wrist were swollen but were neither tender nor warm (Figs. 1 and 2). The right knee was swollen but neither tender nor warm (Fig. 3). While he was able to completely flex and extend the left hip and knee, he was not able to do so on the right side. Passively he could rotate hips 90° pain free. No feet deformity was observed. The rest of his musculoskeletal and systemic examination were unremarkable.

**Fig. 4:**
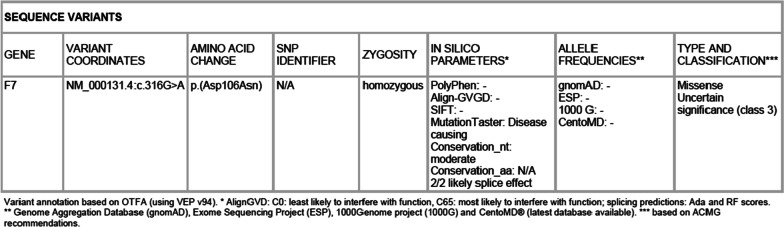
Genetic result summary

Laboratory investigations revealed normal platelet count of 435 × 10^3^/UL (normal 150–450 × 10^3^), hemoglobin 9.67 g/dl (normal ≥ 11.5 g/dl), alanine aminotransferase 13U/L (normal 0–40U/L), albumin 38 g/L (normal 34–54 g/L), and creatinine 43.5 micromol/L (normal 27–62 micromol/L), however, the prothrombin time (PT) reported no coagulation (normal range 11–13 seconds) with normal activated partial thromboplastin time (aPTT) of 39.6 seconds (normal 25–45 seconds). Factor assays routinely available at our facility were done and revealed the following results; factor XIII activity was 155% (normal range 50–150%), factor X was 75% (normal range 68–125%), and von Willebrand factor activity was 202% (normal range 50–160%). Factor VII deficiency (< 1%) (normal 70–156%) was subsequently diagnosed following an extra step taken by sending the sample outside the facility. Genetic testing for this patient, which was sent abroad to CENTOGENE (the rare disease company), identified a homozygous variant of uncertain significance in the FVII gene. The FVII variant causes an amino acid change from aspartic acid to asparagine at position 106 (Fig. 4).

This patient was treated with immediate transfusion of fresh frozen plasma (FFP) since FVII concentrates are not available in our facility. He was also given intravenous vitamin K injection and tranexamic acid tablets. The patient was discharged home 1 week later after bleeding had been arrested and the family was adequately counseled. He is currently not on prophylactic medications due to unavailability of FVII concentrates in our setting, but is following up at his hometown regional referral hospital regularly and receives FFP when needed. The frequency of epistaxis has been dramatically improved and the swelling of the knee and wrist have reduced, which enable him to walk without limping.

## Discussion

FVII deficiency was first described in the medical literature by Alexander *et al*. in 1951 and was referred to as prothrombin conversion accelerator deficiency [7]. This condition is an autosomal recessive disorder, and over 100 mutations, mostly missense mutations, have been identified in the FVII gene located on chromosome 13 [9]. Two types of FVII deficiency have been described, which are type 1 deficiencies resulting from decreased biosynthesis or accelerated clearance, and type 2 abnormalities representing a dysfunctional molecule. In extremely rare instances, FVII deficiency can be acquired as a consequence of severe liver disease, sepsis, or vitamin K deficiency. Certain drugs such as warfarin can also cause acquired FVII deficiency. Acquired FVII deficiency is reported to be more common than the inherited form [9].

Our patient presented with history of recurrent epistaxis and hemarthrosis, which are the most common manifestations of FVII autosomal recessive pattern, affect both sexes, and may skip a generation. The grandfather of our patient, his sister, and his brother were reported to have similar symptoms, while both of his parents were symptom free. The presentation of FVII deficiency in family has been previously reported as was noted by Girolami *et al*. [10]. Age at presentation and positive family history suggest our patient has an inherited rather than acquired form of FVII deficiency.

The PT is prolonged in FVII deficiency and the aPTT is within the reference range in isolated FVII deficiency as seen in our patient. Factor VII gene (F7) is located on chromosome 13q34, 2.8 kb upstream of the FX gene (FX), and contains nine exons encoding the FVII protein circulating in plasma as a 406-amino-acid single chain (50 kDa) [11]. Mutations of FVII gene, which have been characterized in the majority of patients with FVII-deficiency, are very heterogeneous with predominance of missense changes in up to 80% of cases [11].

Management of FVII deficiency associated acute hemorrhage requires FVII replacement therapy and levels of FVII of more than 10% are usually hemostatic, although higher levels may be advisable in severe cases [12, 13]. FVII has a short half-life (3–4 hours), necessitating repeat treatment in all cases with exception of minor bleeding episodes. In the absence of FVII, alternative treatments include fresh frozen plasma, which is least effective because of the volume required to provide adequate FVII replacement. In resource-limited settings, such patients are treated with fresh frozen plasma, vitamin K injection, and tranexamic acid, as in our patient.

## Conclusion

Factor VII deficiency is a rare cause of bleeding disorder, which should be suspected in patients presenting with bleeding who may have family history involving male and female members. Strong consideration should be placed in patients with normal aPTT and platelet count but deranged PT. Efforts should be made to make FVII available, at least in tertiary hospitals.

## Data Availability

Not applicable.

## Abbreviations

FVII: Factor VII
EPI: : Expanded Program of Immunization
PT: : Prothrombin time
aPTT: Activated partial thromboplastin time
